# Surface curvature-directed *in situ* synthesis of ultrathin 2D MOFs on liquid metals for antibacterial applications

**DOI:** 10.1039/d5sc09934j

**Published:** 2026-02-11

**Authors:** Jie Qi, Yihang Zhu, Chen Hang, Hao Tang, Lingmin Zhang, Xingyu Jiang

**Affiliations:** a Guangzhou Municipal and Guangdong Provincial Key Laboratory of Molecular Target & Clinical Pharmacology, The NMPA and State Key Laboratory of Respiratory Disease, School of Pharmaceutical Sciences and the Fifth Affiliated Hospital, Guangzhou Medical University Guangzhou Guangdong 511436 P. R. China zhanglm@gzhmu.edu.cn; b Guangdong Provincial Key Laboratory of Advanced Biomaterials, Department of Biomedical Engineering, Southern University of Science and Technology No 1088, Xueyuan R/d, Xili, Nanshan District Shenzhen Guangdong 518055 P. R. China jiang@sustech.edu.cn

## Abstract

Although promising as hybrid materials for catalysis, antibacterial treatments, and optoelectronics, conventional two-dimensional metal–organic frameworks (MOFs) sheets tend to pile into thick stacks to result in low surface areas. Here, we report a surface curvature-guided *in situ* synthesis strategy that employs gallium-based liquid metal particles (LMPs) as a confined reaction medium for fabricating ultrathin, large-area 2D MOF sheets. By implementing a pre-sonication step in decanol followed by centrifugation before synthesis in DMF, we create conditions that direct MOF growth outward from the curved LMP surfaces, resulting in MOF@LMP microflowers with ultrathin and uniform 2D MOF petals, which we call LOTUS (Liquid-metal Organized Thin Uniform Sheets). These microstructures exhibit exceptional antibacterial efficacy against multi-drug-resistant Gram-positive bacteria, with a minimum inhibitory concentration (MIC) of 2.4 µg mL^−1^, operating through a synergistic mechanism that combines physical disruption, gallium ion release, and reactive oxygen species generation. Biocompatibility assessments further validate their potential for therapeutic applications. Beyond antibacterial applications, our findings establish LMPs as a transformative templating medium for the controlled fabrication of ultrathin 2D MOFs with enhanced functional properties.

## Abstract 2

Liquid-metal curvature guides *in situ* growth of ultrathin MOF petals, yielding a composite with synergistic antibacterial activity against drug-resistant bacteria.

## Graphical abstract

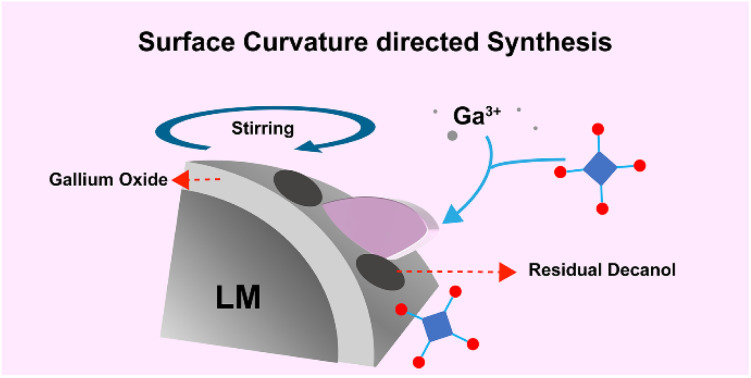

## Introduction

Metal–organic frameworks (MOFs), characterized by their periodic coordination structures and high porosity,^1,2^ have demonstrated broad antimicrobial potential through mechanisms such as metal ion release,^3,4^ antimicrobial ligands,^5^ porous loading of antibacterial agents or gases, photodynamic effects,^6^ physical puncture actions,^7^ and enzyme-like catalysis.^8^ Among these, 2D MOFs have attracted attention for their ability to disrupt bacterial biofilms *via* physical puncture effects.^9^ However, conventional synthesis methods often yield thick, stacked 2D MOFs with limited lateral dimensions. This not only reduces their accessible surface area but also, more critically, diminishes the unique properties inherent to ultrathin, expansive 2D sheets—properties that are vital for diverse applications ranging from electronics (*e.g.*, band gap in 2D semiconductors^10^) to the physical disruption of bacterial membranes. While ligand-modification strategies have been proposed to synthesize ultrathin 2D MOFs,^11–13^ such approaches lack universality and will change the chemical structure of the ligand, potentially compromising other functionalities. Therefore, developing a generalizable method to produce large-area, ultrathin, and non-stacked 2D MOFs remains a significant challenge. Bacterial infections, particularly those caused by antibiotic-resistant Gram-positive bacteria, represent a critical healthcare challenge requiring innovative antimicrobial approaches.^14,15^ Gram-positive bacterial infections lead to severe symptoms that dramatically reduce patient survival rates in intensive care units.^16–18^ Consequently, identifying reliable antibacterial agents effective against multi-drug-resistant bacteria has become an urgent priority for researchers. Numerous nanomaterials, including those composed of metals,^15,19,20^ molecules,^14^ carbon materials,^21^ and metal oxides,^22^ have been introduced to address this pressing issue.

Gallium-based liquid metals (LMs) offer a unique combination of properties that make them promising platforms for antibacterial applications^23^ and 2D material synthesis.^24,25^ These materials have emerged as a prominent class due to their metallic conductivity, biocompatibility, and intrinsic fluidity at room temperature, which endows them with exceptional stretchability and processability.^26–29^ These properties have spurred significant interest in applications such as flexible electronics^30–35^ and biomedical materials.^36–38^ Notably, the high chemical reactivity of gallium enables spontaneous reactions with water in physiological environments, generating gallium ions and hydrogen gas. The released gallium ions exhibit antibacterial effects by disrupting bacterial iron metabolism,^19,20^ while the reduction ability of hydrogen serves therapeutic roles in some diseases.^20^ Furthermore, the atomically smooth surfaces of LMs make them ideal substrates for chemical synthesis, facilitating the growth of large-area, ultrathin two-dimensional materials for diverse applications.^36,37,39^ Additionally, LMPs can serve as dynamic platforms for catalytic metal cluster formation *via* galvanic replacement reactions,^40–42^ offering versatile strategies for engineering antimicrobial LM-based materials. The curvature of a substrate is increasingly recognized as a powerful geometric parameter to direct nanomaterial growth and impart complex, anisotropic functionalities, as exemplified by recent advances in asymmetric hollow structures and patchy colloids.^43–45^

In this work, we aimed to leverage LMPs as a facile and effective template to address the aforementioned synthesis challenge. Here, our surface curvature-guided *in situ* synthesis approach leverages LMPs to grow ultrathin 2D MOFs with enhanced antibacterial properties against multi-drug-resistant bacteria. It is noteworthy that the targeted 2D MOF, Ga-TCPP (tetrakis(4-carboxyphenyl)porphyrin), is a known material whose structure and conventional synthesis have been previously reported.^46^ Building on the concept of MOF synthesis using metal ions released by a LM,^47^ and distinct from the conventional homogeneous synthesis of Ga-TCPP, we report the development of versatile antibacterial microflowers through the *in situ* growth of 2D MOFs (Ga-TCPP) on LMPs (LOTUS). By using LMPs as a dynamic, self-templating substrate for the *in situ* growth of 2D MOFs, we achieve ultrathin Ga-TCPP MOF sheets distributed evenly across the LMP surface. Through careful control of reaction time, we observed the entire growth process of these 2D MOFs. X-ray photoelectron spectroscopy (XPS) analysis confirms the absence of coordination between the porphyrin center and gallium ions (distinguishing our approach from previously reported thin 2D MOF synthesis strategies^48^), demonstrating that the LMP reaction medium enables the synthesis of ultra-thin 2D MOFs while preserving their native chemical structure. The prepared LOTUS demonstrated remarkable efficacy in eliminating both conventional and multi-drug-resistant Gram-positive bacteria through a synergistic antibacterial mechanism involving reactive oxygen species (ROS), ROS-independent oxidation processes, physical damage inflicted by the 2D MOFs, and the inherent antibacterial properties of Ga^3+^. Overall, our LOTUS constructs demonstrate high antibacterial efficacy coupled with excellent biocompatibility, establishing surface curvature-guided *in situ* synthesis on LMPs as a promising approach for developing functionally enhanced 2D MOFs with unique properties.

## Results and discussion

### Preparation and characterization of LOTUS

**Scheme 1:**
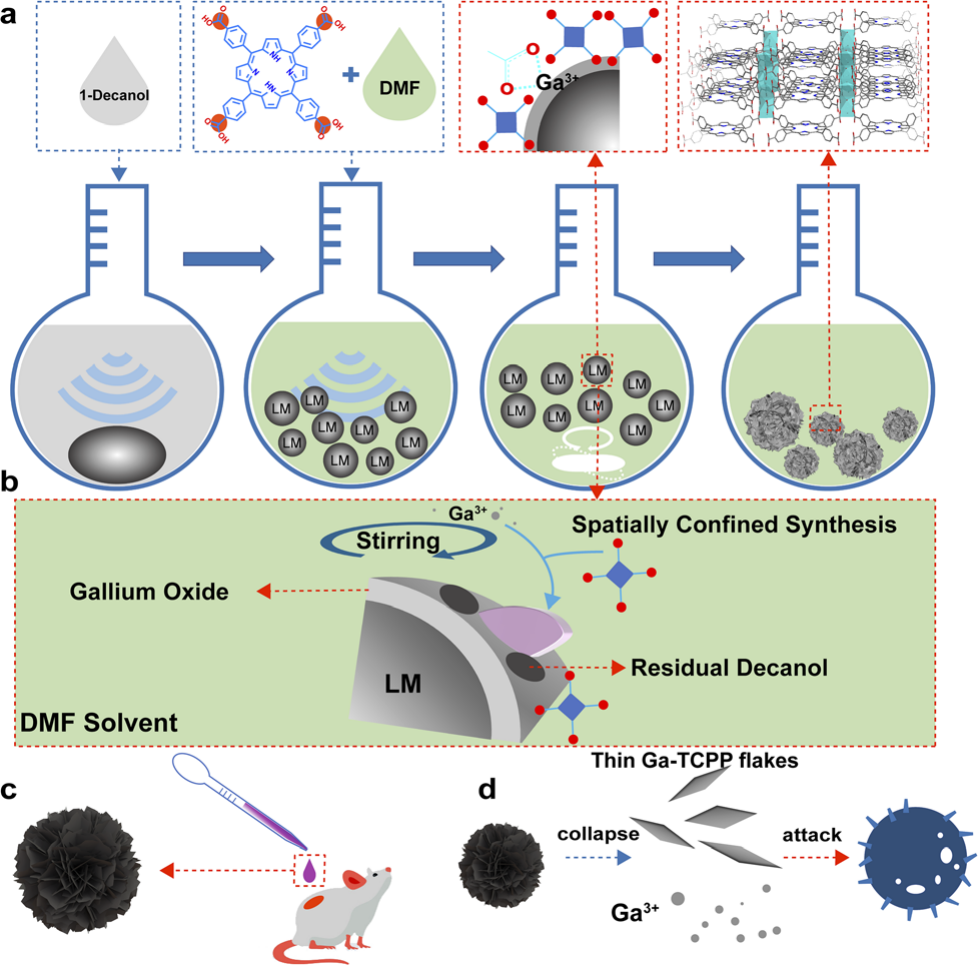
Schematic illustration of the preparation of LOTUS and its antibacterial process. (a) Schematic illustration of the method to prepare LOTUS. (b) Spatially confined synthesis of MOFs on the LMPs. (c) LOTUS applied as an antibacterial agent for curing antibacterial infection. (d) LOTUS collapse into thin Ga-TCPP flakes and Ga ions and work together to kill the bacteria.

As demonstrated in Scheme 1a, we first sonicated a LM in 1-decanol and the LMPs were precipitated and transferred into the *N*,*N*-dimethylformamide (DMF) solution of TCPP. This process is used to control the growth of MOFs on the LMPs. During this process, TCPP can attach onto LMPs which came from the coordination of Ga ions and carboxyl group. This surface modification is confirmed by TEM EDS (Fig. S1), and we can find the distribution of *N* (which is contained in TCPP) on the LMPs. The size distribution of LMPs under different sonication times is characterized by SEM (Fig. S2). Along with the heating and stirring, the Ga ions were released from the LMPs and coordinated with TCPP, and Ga-TCPP 2D MOFs were synthesized on the surface of LMPs, resembling microflowers. As a typical MOF, the resulting Ga-TCPP possesses an intrinsic microporous structure, with a reported high surface area (1150–1400 m^2^ g^−1^).^46^ Consequently, the as-synthesized LOTUS composite may retain DMF solvent within these pores. To ensure complete removal of this toxic solvent for subsequent biological applications, a rigorous purification protocol was essential. The as-synthesized product was then thoroughly purified *via* sequential solvent washing and dialysis to remove the DMF solvent. Fourier-transform infrared (FTIR) spectroscopy confirmed the absence of residual DMF in the final LOTUS material (Fig. S3).^49^ Thermal stability was confirmed by TGA (Fig. S4). As depicted in Scheme 1b, the conjugated growth process is driven by decanol on the surface of LMPs, directing the outward growth of MOFs. Continuous bead stirring during this process supplies gallium ions essential for MOF synthesis, a mechanism further elucidated in the following section.

**Fig. 1:**
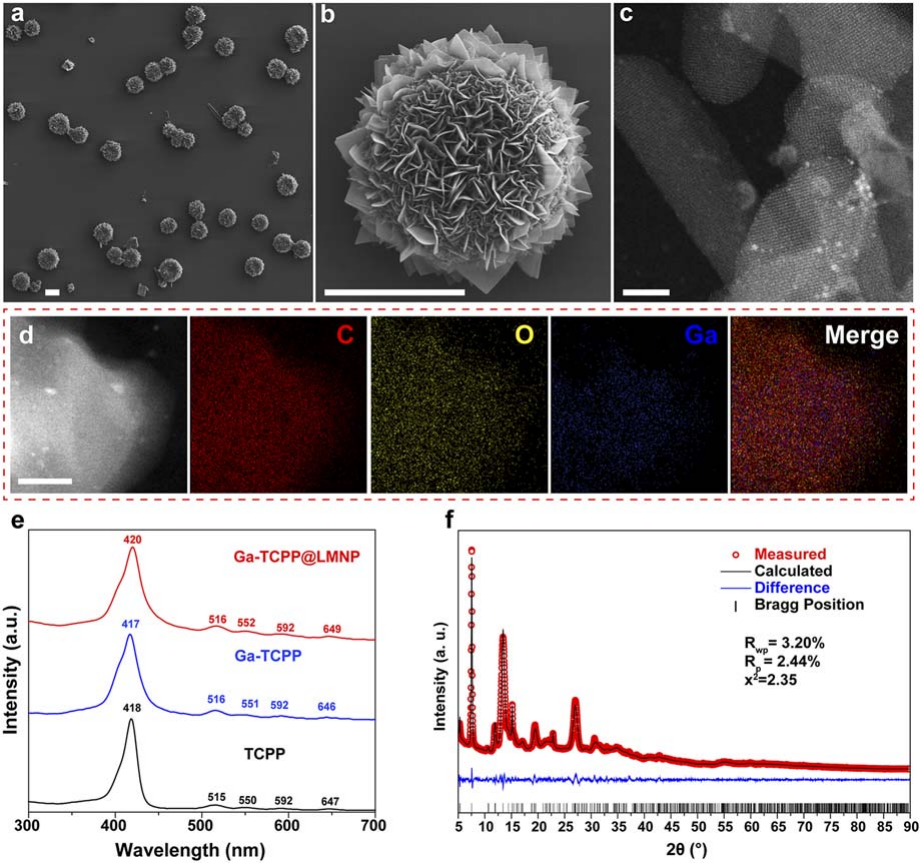
Characterization of the LOTUS. (a) SEM image of LOTUS and (b) the magnified image; the scale bar is 5 µm. (c) TEM image of the MOF flake on LOTUS; the scale bar is 20 nm. (d) TEM EDS mapping image of LOTUS; the scale bar is 50 nm. (e) UV-vis spectrum of Ga-TCPP MOFs, LOTUS and pure TCPP in ethanol. (f) Result of the Rietveld refinement of LOTUS.

The morphology of LOTUS was characterized by SEM (Fig. 1a and b) and some free MOF flakes were also observed. From the SEM images, we can find that the Ga-TCPP MOFs on the LMPs exhibit uniform, ultrathin thicknesses and all grow outward perpendicular to the surface of LMPs. The size distribution of LMPs before and after the synthesis of MOFs is characterized by DLS (Fig. S5). The morphology of the Ga-TCPP MOF flakes was characterized by TEM (Fig. 1c) and the element composition was confirmed by EDS mapping (Fig. 1d). From the TEM image, we can find the Ga-TCPP MOFs are ultrathin. Directly synthesized Ga-TCPP MOFs have a large thickness and a nano-scale size which came from the interaction (face-to-face and edge-to-edge) between different MOFs layers. To highlight the advantage of our method in preventing layer stacking, we compared LOTUS with Ga-TCPP MOFs synthesized *via* conventional solvothermal methods. As shown in Fig. S6, the conventional product exhibited severe stacking: densely packed nanosheets and thick micrometer-sized aggregates. From the UV-vis spectrum (Fig. 1e), the four Q bands of the Ga-TCPP MOFs and the Ga-TCPP@LM were unchanged which indicates that there is no central coordination. Without the central coordination to decrease the interaction between TCPP molecules, these ultra-thin 2D MOFs sheets may come from the restricted growth process on the curved surface of LMPs and we will explore it in the next section. The crystal structure of the composite was confirmed by powder X-ray diffraction (XRD). The XRD pattern of LOTUS (Fig. 1f) shows the characteristic diffraction peaks of the Ga-TCPP MOF. To rigorously identify the MOF phase, the pattern was analyzed *via* Rietveld refinement using the reported CIF file for Ga-TCPP^46^ within the GSAS-II software.^50^ The refinement, which excluded the non-crystalline LM background, converged with a low goodness-of-fit (*R*_wp_ = 3.2%, *R*_p_ = 2.44%, and *x*^2^ = 2.35), confirming that the MOF component in LOTUS possesses an identical crystal structure to the reference Ga-TCPP framework. This result provides definitive evidence for the successful *in situ* formation of the target MOF on the LMP surface.

### The growth process of LOTUS and the different growing positions of Ga-TCPP MOFs

**Fig. 2:**
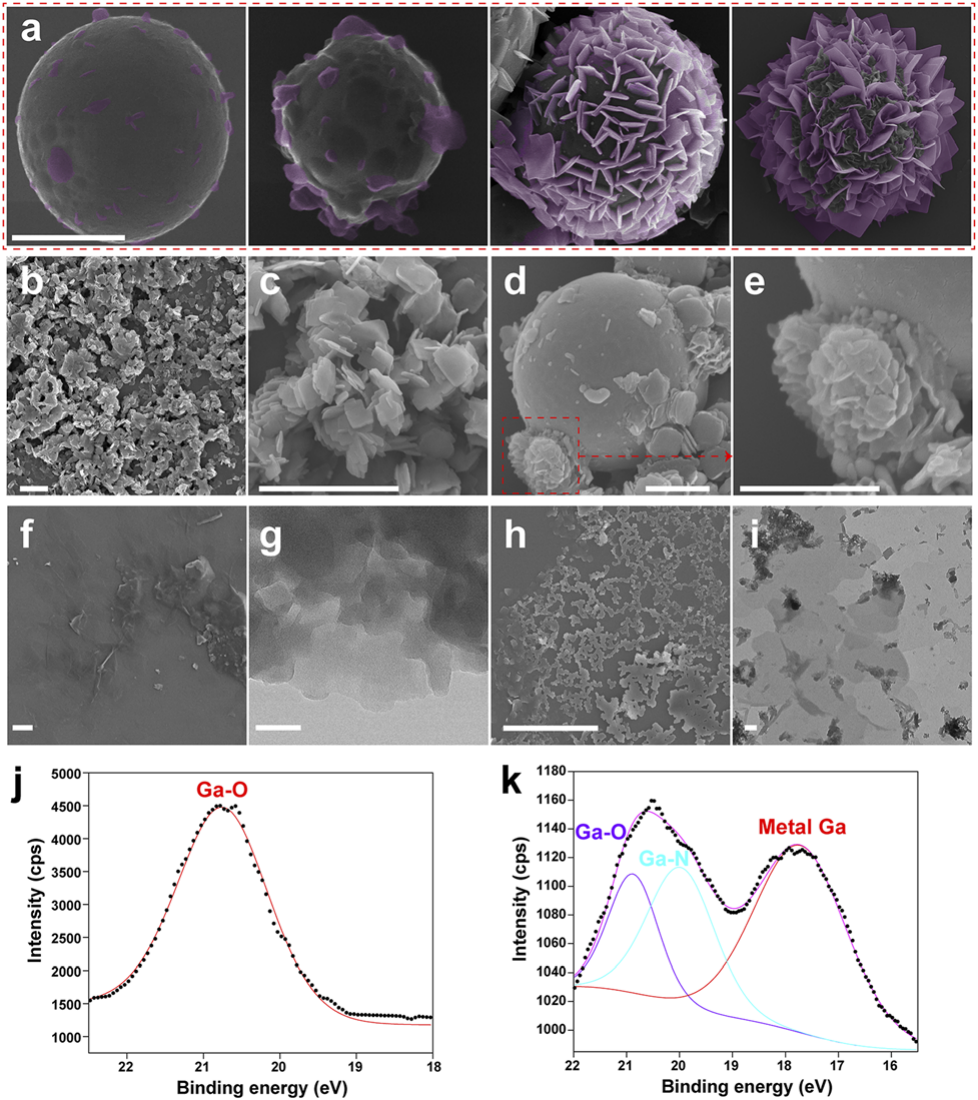
The growth process of the LOTUS. (a) SEM images of LOTUS which are in different growth stages, the reaction time is 2 h, 4 h, 8 h and 10 h, from left to right; the scale bar is 5 µm. (b) and (c) SEM images of Ga-TCPP MOFs synthesized without the sonication in decanol, (c) is the enlarged image of (b); the scale bar is 2 µm. (d) and (e) SEM images of Ga-TCPP MOF modified LMPs synthesized without the sonication in decanol, (e) is the enlarged image of the red dotted frame. (f) SEM image of the Ga-TCPP MOFs synthesized with the sonication in decanol, the MOFs are separated from LMPs by acid treatment; the scale bar is 10 µm. (g) TEM image of the thin Ga-TCPP MOFs; the scale bar is 50 nm. (h) SEM image of the LOTUS treated by LB medium overnight; the scale bar is 5 µm. (i) TEM image of the LOTUS treated with LB medium; the scale bar is 500 nm. Ga 3d XPS spectra of (j) pristine Ga-TCPP MOFs and (k) LOTUS.

For further investigation towards the growth process of MOF sheets on the LMPs and explore the mechanism of synthesizing ultra-thin MOF sheets, we used SEM to characterize the morphology of LOTUS with different reaction times (Fig. 2a). We have used false color to highlight the emerging MOF sheets. We can find that some small sheets appear first and they grow bigger when some other sheets start to appear. The reaction time of these four stages is 2 h, 4 h, 8 h, and 10 h respectively. We can find that in the initial stage of growth, the MOF sheets grow mostly perpendicular to the particle surface.

Pre-sonication in decanol enables the growth of ultra-thin Ga-TCPP MOF sheets perpendicularly on LMPs, whereas its absence leads to thicker, parallel sheets and solution-based MOF formation, highlighting the critical role of solvent choice and preparation conditions in tailoring MOF synthesis. To further understand the role of solvent polarity in this interfacial process, control experiments were performed using a series of alcohols (methanol, ethanol, and 1-hexanol) for the pre-sonication step. The resulting MOF morphologies, summarized in Fig. S7, show a clear progression: high-polarity alcohols (methanol and ethanol) led to curled, aggregated, or small thick sheets, while lower-polarity 1-hexanol yielded structures morphologically similar to an intermediate stage of LOTUS growth (Fig. 2a). This trend supports the view that a less polar alcohol, such as decanol, facilitates the interfacial kinetic control necessary for the confined, outward growth achieved in our synthesis. DMF is widely utilized as a solvent in MOF synthesis due to its high boiling point, templating effects, and favorable acid–base properties.^51^ However, the high polarity of decanol renders it unsuitable as a solvent for MOF synthesis; inclusion of decanol enables kinetic control, directing MOF growth outward from LMPs. Without pre-sonication in decanol, Ga-TCPP MOFs also form in solution (Fig. 2b and c) when the LM is sonicated directly in DMF and subsequently mixed with TCPP solution. Under these conditions, MOF sheets predominantly grow parallel to the particle surface (Fig. 2d and e) and are significantly thicker than those grown perpendicularly on LMPs. Additionally, some MOF sheets aggregate during growth, forming spherical structures. This behavior results from variations in Ga-TCPP MOF growth rates: DMF enhances TCPP deprotonation, promoting MOF synthesis, whereas residual decanol impedes this process. Comparing Ga-TCPP MOFs grown on LMPs with pre-sonication in decanol (Fig. 2f and g; sheets collected *via* continuous shaking) to those without pre-sonication reveals that the latter produces thicker, smaller-area sheets.

To elucidate how the curvature of the LMP substrate governs MOF morphology, we performed synthesis under controlled conditions by varying the substrate geometry. First, LOTUS was synthesized using LMPs of different sizes (by controlling the sonication time), which provide varying radii of curvature (Fig. S8). When very small LMPs (diameter < 1 µm) were used, the limited surface area led to overcrowded nucleation. The subsequently growing MOF sheets extensively overlapped and interfered with each other, resulting in smaller, thicker, and less uniform petals. In contrast, using larger LMPs (diameter > 5 µm) provided sufficient space for individual nuclei to develop independently, yielding the larger, thinner, and uniform 2D sheets characteristic of the optimal LOTUS morphology.

Second, as a critical control, we performed the synthesis on a flattened LM film, which presents a near-zero curvature surface (Fig. S9). In stark contrast to the success of vapor-phase 2D material synthesis on such films,^52^ our solution-phase coordination reaction on the flat film yielded severely stacked and entangled MOF aggregates, with no formation of discrete, outward-oriented sheets. This result highlights a fundamental difference between reaction regimes and underscores that a particulate form with finite curvature is indispensable in our system. It provides the necessary spatial confinement to direct nucleation and guide outward, anisotropic MOF growth, effectively preventing the face-to-face stacking that prevails in homogeneous solutions or on flat surfaces. These experiments demonstrate that the surface curvature of LMPs is a critical design parameter. An optimal curvature, achieved with LMPs of adequate size, is essential to template the growth of the ultrathin, large-area 2D MOF sheets that constitute the LOTUS architecture. This aligns with the broader principle that curved interfaces can direct anisotropic growth and assembly.^45,53^

Collectively, these results delineate a clear formation mechanism for LOTUS. The slow release of Ga^3+^ from LMPs seeds MOF nucleation directly on the surface. The residual medium-polarity solvent from pre-sonication moderates interfacial kinetics, confining growth to the template. Crucially, the finite curvature of adequately sized LMPs then spatially directs this confined growth outward, preventing face-to-face stacking and allowing the Ga-TCPP sheets to expand into large, thin, and discrete petals.

We treated the LOTUS with LB medium and the LOTUS collapse into tiny gallium nanoparticles and small Ga-TCPP MOF sheets (Fig. 2h), which reveals the morphological changes of LOTUS after interacting with bacteria. The morphology of collapsed LOTUS is characterized by TEM (Fig. 2i), and we used TEM EDS to characterize the element composition of the collapsed LOTUS (Fig. S10). The TEM EDS images show that the Ga-TCPP MOFs sheets collapse into small sheets whose central part is thinner than the other part, and some tiny gallium nanoparticles (around 10 nm) are attached to these small sheets.

We used XPS to characterize the chemical composition in the surface of pristine Ga-TCPP MOFs (Fig. 2j) and LOTUS (Fig. 2k). The Ga 3d spectrum of pristine Ga-TCPP MOFs (Fig. 2j) shows the existence of the Ga–O bond which comes from the coordination bond between Ga and the carboxyl of TCPP. The Ga 3d spectrum of LOTUS (Fig. 2k) shows the existence of Ga–O, Ga–N and metallic gallium. The signal of Ga–N may come from the central coordination between TCPP and Ga. This coordination does not appear in the UV-vis spectrum, and it means that only a few Ga atoms are centrally coordinated.

### Exploration of the strategy with a 3D Ga-MOF

To preliminarily probe the generality of our surface curvature-guided synthesis beyond the 2D Ga-TCPP system, we applied a similar protocol to a reported three-dimensional (3D) Ga-based MOF.^54^ As shown in Fig. S11, the 3D MOF, which typically forms needle-like microcrystals in conventional solution synthesis, instead yielded shorter, rod-shaped structures uniformly distributed on the LMP surface under our conditions. This morphological change suggests that the slow, sustained release of Ga^3+^ from the liquid metal surface can promote more homogeneous nucleation and growth, leading to improved morphological uniformity. However, due to the intrinsically isotropic 3D coordination network, the resulting structures did not exhibit the anisotropic, layered growth necessary to form the ultrathin 2D “petal” or nanoflower-like architecture characteristic of the LOTUS system. This comparison underscores that while the LMP platform can modulate growth kinetics and uniformity, the formation of the distinct 2D microflower morphology is closely tied to the layered crystal habit of the 2D Ga-TCPP MOF itself.

### Bactericidal activity of LOTUS *in vitro* and preliminary mechanism exploration on the antibacterial activity

We evaluated the *in vitro* bactericidal activity of LOTUS. Given that the porphyrin core of the TCPP linker is a classic photosensitizer capable of generating ROS under light irradiation—a well-established antimicrobial mechanism^6^—we sought to assess whether this photodynamic effect could enhance the antibacterial performance of LOTUS. Therefore, experiments were conducted both in the presence and absence of light. Six bacterial isolates including Gram-positive (*S. aureus* and *S. epidermidis*) and Gram-negative (*E. coli* and *P. aeruginosa*) as well as the corresponding MDR isolates of the Gram-positive species were chosen as the model bacteria. We tested the minimum inhibition concentrations (MICs) of LOTUS towards these bacterial isolates with and without light (Table 1). The MIC values listed represent the lowest concentration that completely inhibited bacterial growth across three independent experiments. LOTUS has excellent antibacterial properties towards these Gram-positive bacteria: MIC without light is twice larger than the MIC with light, indicating that the irradiation of light can enhance the antibacterial activity of LOTUS. The control experiments provide further insight into the origin of LOTUS's high efficacy. As shown in Table 1, neither pure TCPP nor LMPs alone showed significant antibacterial activity within the tested concentration range (MIC ≥ 62.5 µg mL^−1^ for all strains). The physical mixture of TCPP and LMPs (TCPP + LMPs) showed only marginal improvement (for *S. aureus* and MRSA), with MIC values remaining high and substantially above those of LOTUS. These results underscore the critical importance of the *in situ* synthesized microstructure. The poor performance of the individual components and their simple mixture indicates that neither the ligand nor the LMPs alone—nor their physical association—can recapitulate the antibacterial potency of LOTUS. This suggests that the ultrathin, and radially oriented 2D MOF petals grown on LMPs are essential for achieving high antibacterial activity, likely due to their large specific surface area, enhanced physical piercing capability, and optimized interfacial contact with bacteria. Notably, the TCPP + LMPs mixture did show slightly lower MIC values against *S. aureus* and MRSA compared to the individual components, suggesting a synergistic interaction between Ga^3+^ released from LMPs and the TCPP ligand. This synergy arises from the combined effect of Ga^3+^ disrupting bacterial iron metabolism and the iron ion capture properties of the porphyrin (similar structure with hemin at its central structure, making TCPP a suitable carrier for ion exchange^55,56^).

**Table 1:** Minimum inhibitory concentration (MIC, µg ml^−1^) values of LOTUS-light and -dark against Gram-negative bacteria (*P. aeruginosa* and *E. coli*), Gram-positive bacteria (*S. aureus* and *S. epidermidis*), and their drug-resistant counterparts (MRSA and MRSE)

	MIC (µg ml^−1^)					
	*P. aeruginosa*	*E. coli*	*S. aureus*	*S. epidermidis*	MRSA	MRSE
LOTUS-light	38.4	76.8	2.4	4.8	2.4	2.4
LOTUS-dark	38.4	76.8	4.8	9.6	4.8	4.8
TCPP-light	>250	>250	>250	62.5	>250	>250
LMPs-light	>250	>250	>250	>250	>250	>250
TCPP + LMPs-light	>250	>250	250	62.5	250	>250

**Fig. 3:**
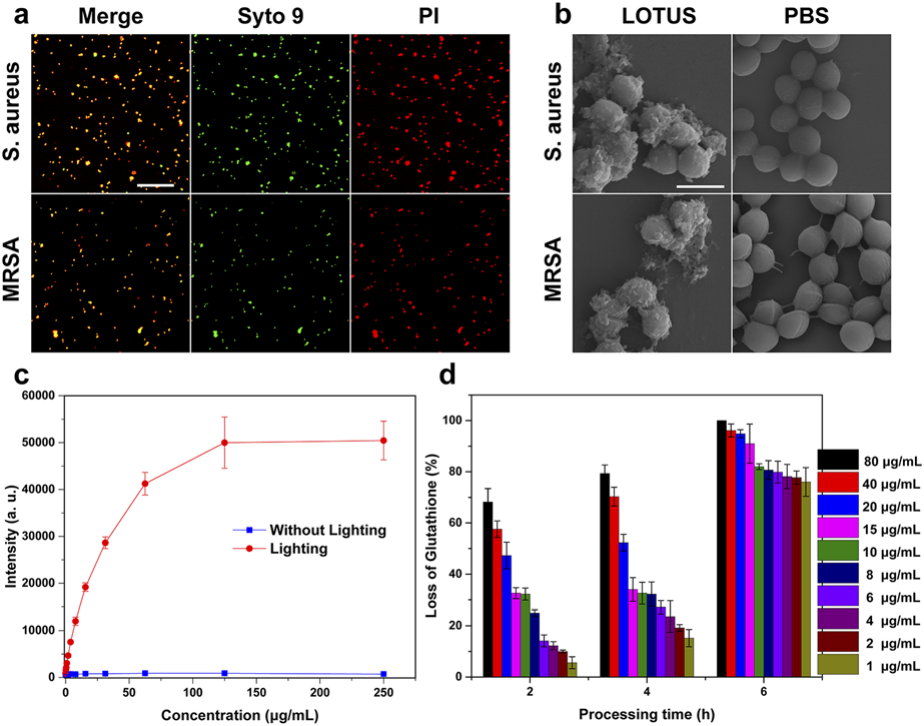
Exploration of the bactericidal mechanism of LOTUS. (a) Confocal images of *S. aureus* and MRSA treated with LOTUS and stained with PI and Syto 9; the scale bar is 50 µm. (b) SEM images of the morphology of bacteria with the treatment of LOTUS; the scale bar is 1 µm. (c) The concentration of reactive oxygen species generated by LOTUS under different conditions. (d) Oxidation of glutathione without ROS by LOTUS of different concentrations.

We employed confocal microscopy to examine whether LOTUS can break the integrity of bacterial outer membranes (Fig. 3a) with the bacteria stained with Syto 9 and PI. The green fluorescence signal of Syto 9 can be obtained from live and dead bacteria while the red signal of PI can only be obtained from the dead bacteria. The outcome shows the powerful bactericidal properties of LOTUS. In order to further explore the broken membrane of bacteria, we used SEM to characterize the morphology of the *S. aureus* and multi-drug-resistant *S. aureus* (MRSA) treated with LOTUS solution and PBS (phosphate buffered saline). The LOTUS collapsed into MOF pieces in the culture medium and attached to the surface of the bacteria (Fig. 3b). We can observe that the membrane was disrupted and surrounded with small MOF flakes. This provides direct evidence for a physical disruption mechanism, where the sharp, ultrathin MOF sheets can pierce and damage bacterial membranes.^57–59^

In order to investigate the antibacterial mechanism of LOTUS, we tested the ROS production properties and the ROS independent oxidation properties of LOTUS. We used DCFH-DA as the indicator and the result (Fig. 3c) shows that the photoluminescence intensity increased greatly under the irradiation of a daylight lamp for 10 min. This confirms the light-induced ROS generation, a key photodynamic antibacterial pathway associated with the porphyrin ligand.^6,60,61^ The light-induced ROS production can explain the different antibacterial efficiencies of LOTUS with and without lighting (Table 1). We also used the loss of glutathione to characterize the ROS independent oxidation properties of LOTUS, and the result (Fig. 3d) shows that the LOTUS can oxidize the glutathione without lighting and higher concentration leads to a higher oxidation efficiency. This demonstrates a ROS-independent oxidative stress pathway contributing to the dark-condition antibacterial activity.^62,63^ This result explains the high antibacterial properties of LOTUS under the dark condition.

### Comparison of antibacterial performance

The potent antibacterial activity of LOTUS against Gram-positive bacteria (MIC as low as 2.4 µg mL^−1^) is notable among Ga-containing materials. A meaningful comparison considers both the effective dose and the material's physical scale. For instance, a LM-based composite of similar size demonstrated antibacterial action at 100 µg mL^−1^, relying on an external field to induce bactericidal morphology.^23^ In contrast, LOTUS achieves significant efficacy at a dose over 40 times lower without such external activation. When compared to a nanoscale Ga-MOF (∼200 nm spheres) with an MIC of ∼1 µg mL^−1^,^64^ LOTUS, despite its much larger size (and consequently lower particle count at the same mass concentration), attains a comparable MIC. This indicates an exceptionally high intrinsic activity per particle, stemming from the synergistic effects of physical disruption, Ga^3+^ release, and ROS generation unique to the LOTUS architecture.

### The biocompatibility of LOTUS

**Fig. 4:**
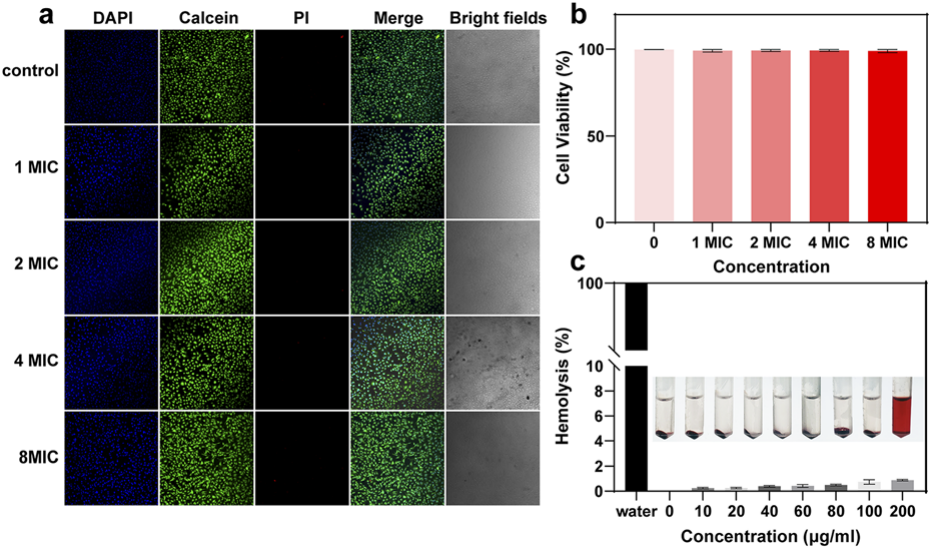
The biocompatibility test of LOTUS. (a) Confocal images of HUVECs which were cultured with LOTUS of different concentrations. (b) The cell viability after being cultured with LOTUS; data are presented as mean ± SD from three independent experiments. (c) Hemolysis assay of LOTUS; hemolysis percentage was determined by measuring hemoglobin release at 540 nm. The absorbance of the sample supernatant was normalized against the negative control (0% hemolysis, in PBS) and positive control (100% hemolysis, in DI water) using the formula: % hemolysis = [(OD_sample_ − OD_negative_)/(OD_positive_ − OD_negative_)] × 100.

Apart from the antibacterial properties, biocompatibility is important for the bio-application of LOTUS. We cultured HUVECs with LOTUS at different concentrations (1× to 8× MIC) and assessed viability using a Live/Dead assay. The confocal microscopy images (Fig. 4a) showed minimal red fluorescence (dead cells) across all groups, visually indicating low cytotoxicity and high biocompatibility. To quantify this effect, cell viability was measured and statistically analyzed (Fig. 4b). Data are presented as mean ± standard deviation (SD) from three independent experiments. No statistically significant differences in cell viability were observed among the concentration groups as determined by one-way analysis of variance (ANOVA), confirming the absence of concentration-dependent cytotoxicity within the tested range. We further evaluated the hemocompatibility of LOTUS *via* a hemolysis assay (Fig. 4c). The results demonstrated that LOTUS caused negligible hemolysis, with no significant damage to rat erythrocytes even at a concentration of 200 µg mL^−1^. According to the data above, the LOTUS is highly biocompatible and suitable for the following *in vivo* experiment.

### Wound healing evaluation with the LOTUS

We tested the therapeutic effect of LOTUS on *S. aureus* and MRSA infected wounds. Skins can behave as the vital barrier to effectively prohibit infection caused by the invasion of microorganism. We built wound models on the back of rats which were infected with *S. aureus* and MRSA to evaluate the healing efficiency. Sixteen rats were divided into two groups (eight for each), and infected with *S. aureus* and MRSA, respectively. For every rat, three areas of dorsal skin of the same size were excised and exposed to the bacteria. After 30 min, for every rat, 100 µL LOTUS solution was dripped onto one wound, and 100 µL PBS was dripped onto the other two wounds, respectively as the controls.

**Fig. 5:**
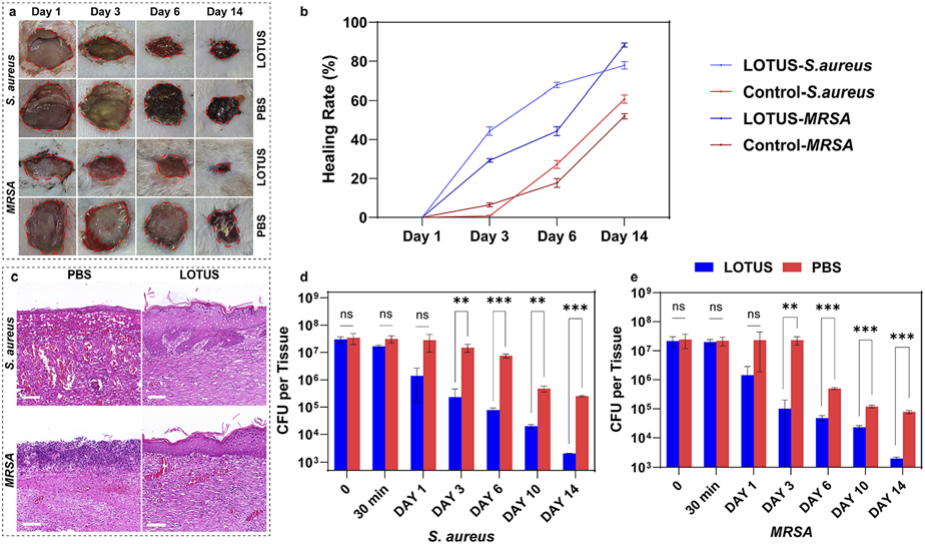
Wound healing evaluation of LOTUS. (a) Photographs of rat wounds infected with *S. aureus* or MRSA under the treatments of PBS and LOTUS. (b) The healing rate of the wounds under different treatments, 
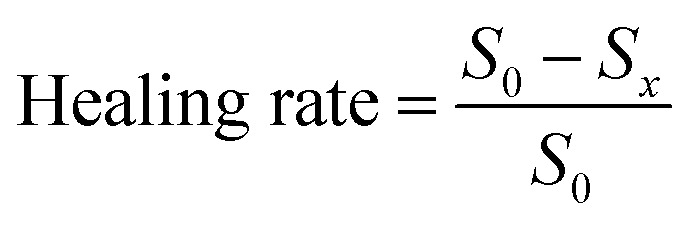
 (*S*_0_: wound area at day 0; *S*_*x*_: wound area at day *x*). (c) H&E staining of rat wounds infected with *S. aureus* or MRSA under different treatments at intervals of 14 days; the scale bar is 100 µm. (d) The number counts of bacteria removed from *S. aureus* infected wounds, colony forming unit (CFU). (e) The number counts of bacteria removed from MRSA infected wounds, (**P < 0.01 and ***P < 0.001).

Wound healing was monitored photographically over time (Fig. 5a). To quantitatively assess healing progression, the wound healing rate was calculated as (*S*_0_ − *S*_*x*_)/*S*_0_, where *S*_0_ is the initial wound area and *S*_*x*_ is the area on day *x*. Data from three parallel wounds per condition were analyzed (Fig. 5b). The LOTUS-treated wounds exhibited markedly accelerated closure, with healing rates on Day 3 and Day 6 more than double those of the PBS-treated controls.

Histological analysis of the wound tissue after 14 days *via* hematoxylin and eosin (H&E) staining (Fig. 5c) further confirmed the enhanced healing. LOTUS-treated wounds displayed complete re-epithelialization and well-organized, uniformly dispersed collagen fibers, features that were notably absent in the control wounds.

Bacterial burden in the wounds was quantitatively evaluated by colony forming unit (CFU) counts (Fig. 5d and e). Statistical analysis (two-way ANOVA with Tukey's *post hoc* test) revealed that LOTUS treatment drastically reduced the bacterial load compared to PBS controls. For *S. aureus*-infected wounds, CFU counts were significantly lower in the LOTUS group on Day 3 (**p < 0.01) and Days 6 and 14 (***p < 0.001). Similarly, in MRSA-infected wounds, LOTUS treatment resulted in significantly lower bacterial counts on Day 3 (**p < 0.01) and on Days 6, 10, and 14 (***p < 0.001).

Collectively, these results demonstrate that LOTUS not only potently reduces wound bacterial burden but also significantly promotes the structural regeneration of infected skin.

To evaluate the anti-infective potential of LOTUS, we established a murine auricular infection model comparing therapeutic outcomes between experimental and control groups (Fig. S12). Intradermal injection (20 µL) containing either PBS-mixed (control) or LOTUS-formulated (1 × MIC, 4.8 µg mL^−1^) bacterial suspensions (10^5^ per CFU) revealed distinct pathophysiological responses through photoacoustic monitoring (Fig. S12a). Quantitative analysis demonstrated that control subjects developed pronounced inflammation within 4 hours post-inoculation, manifesting substantial increase in vascular density and total hemoglobin concentration relative to the original state (Fig. S12b and c). Conversely, LOTUS treatment effectively preserved vascular architecture despite injection-induced edema, with concurrent reductions in both vascular density and hemoglobin concentration, suggesting mitigated inflammatory progression.

## Conclusions

In conclusion, our study demonstrates the successful *in situ* growth of 2D MOF sheets on LMPs, offering a surface curvature-guided approach to synthesizing ultra-thin, large-area 2D MOF sheets. The method, involving pre-ultrasonication treatment in decanol and *in situ* growth on the surface of particles, facilitates a confined reaction that results in thinner and larger MOF sheets compared to the traditional homogeneous liquid phase reaction. The resulting microflowers exhibit potent antibacterial activity against Gram-positive bacteria, with a MIC of 2.4 µg mL^−1^. The material's multifaceted antibacterial mechanisms, including ROS production, ROS independent oxidation, and the release of antibacterial gallium ions, are further enhanced by the physical inhibition provided by the sharp MOF layers. Our findings, validated through *in vivo* and *in vitro* experiments, underscore the therapeutic potential and biocompatibility of LOTUS in treating infected wounds. This work not only advances the field of antibacterial materials but also provides a new strategy for the synthesis of advanced 2D MOF materials, paving the way for future innovations in materials science.

## Ethical statement

All animal experiments were performed in compliance with the relevant laws and institutional guidelines. All procedures for animal studies were conducted according to the approved protocols by the Shenzhen Advanced Animal Study Service Center (Approval No. AASC200615M).

## Author contributions

J. Q. designed the experiments. J. Q., Y. Z., C. H. and H. T. performed the experiments and analyzed the data. J. Q., L. Z., and X. J. prepared the manuscript. L. Z. and X. J. supervised the whole project. All authors have approved the final version of the manuscript.

## Conflicts of interest

There are no conflicts to declare.

## Supplementary Material

SC-OLF-D5SC09934J-s001

## Data Availability

The data supporting this article have been included as part of the supplementary information (SI). Supplementary information: detailed experimental procedures and additional characterization figures referenced in the main text. See DOI: https://doi.org/10.1039/d5sc09934j.
