# Supplementary material for: Surface curvature-directed in situ synthesis of ultrathin 2D MOFs on liquid metals for antibacterial applications
Source: Chem Sci. 2026 Feb 11;17(13):6746–54. doi: 10.1039/d5sc09934j (PMC12893118; doi:10.1039/d5sc09934j)
Supplement: SC-017-D5SC09934J-s001 [file SC-017-D5SC09934J-s001.pdf]

Supplementary Information (SI) for Chemical Science.
This journal is © The Royal Society of Chemistry 2026

Supporting Information for:

Surface Curvature-Directed In-Situ Synthesis of Ultrathin 2D MOFs on Liquid Metal for Antibacterial Applications

Jie Qi,[a,b] Yihang Zhu,[b] Chen Hang,[b] Hao Tang,[b] Lingmin Zhang,*[a,b] and Xingyu Jiang*[b]

[a] Guangzhou Municipal and Guangdong Provincial Key Laboratory of Molecular Target & Clinical Pharmacology, the NMPA and State Key Laboratory of Respiratory Disease, School of Pharmaceutical Sciences and the Fifth Affiliated Hospital, Guangzhou Medical University, Guangzhou, Guangdong, 511436, P. R. China

[b] Shenzhen Key Laboratory of Smart Healthcare Engineering, Guangdong Provincial Key Laboratory of Advanced Biomaterials, Department of Biomedical Engineering. Southern University of Science and Technology, No 1088, Xueyuan R/d, Xili, Nanshan District, Shenzhen, Guangdong, 518055, P. R. China

Corresponding Author: Prof. Xingyu Jiang, Prof. Lingmin Zhang

E-mail: jiang@sustech.edu.cn, zhanglm@gzhmu.edu.cn

Experimental Section

Materials and Devices

Unless otherwise noticed, all relevant reagents are purchased on Sigma-Aldrich, MACKLIN and use without any pretreatment. Eutectic gallium indium (EGaIn) is purchased from Beijing Hawk Technology Co., Ltd, China. Cell live/dead kit are from Invitrogen, US.

All sonication was performed by a sonicator (Scientz, Scientz-IID). Scanning electron microscopy (SEM) images and energy dispersive X-ray (EDX) were obtained using a Hitachi-SU8220 equipped with EDAX genesis 2000 XMS accessory. X-ray

photoelectron spectroscopy (XPS) spectrum was performed on ESCALAB 250Xi (Thermo Fisher). UV-vis spectrum was recorded with a UV2450 spectrophotometer (Shimadzu). All electrochemical tests were performed on the electrochemical workstation CH 602E (CH Instruments, China). Cell fluorescence images were obtained by a confocal microscopy (Zeiss LSM 710). Inductively coupled plasma mass spectrometry (ICP-MS) for Ga ions detection were performed on NexION 300X (Perkinelmer). All photographs were recorded with a Nikon D90 camera.

Bacteria, Cells, and Animals

Escherichia coli (*E. coli*, ATCC 25922), *Klebsiella pneumoniae* (*K. pneumoniae*, ATCC 700603), *Acinetobacter baumanii* (*A. baumanii*, ATCC 19606), *Pseudomonas aeruginosa* (*P. aeruginosa*, ATCC 27853), *Staphyloccocus aureus* (*S. aureus*, ATCC 29213), *Staphyloccocus epidermidis* (*S. epidermidi*, ATCC 12228) were purchased from Beijing Runzekang Biotechnology (China). Clinical isolates, including multidrug resistant (MDR) *S. aureus* (*MRSA*), methicillin-resistant *S. epidermidis* (*MRSE*) were from local hospitals. Bacteria strains are grown in LB and LB agar plates at 37 ^{o}C. Human umbilical vein endothelial cells (HUVEC) cell lines were cultured in Dulbecco's Modified Eagle Media (DMEM, Gibco, USA) in the absence of 10 % (v/v) fetal bovine serum (FBS, Gibco, USA) and 1 % (v/v) penicillin-streptomycin (PS, Gibco, USA) in the cell incubator (37 ^{o}C, 5 % CO_2). Animals, including female balb/c mice and female Sprague-Dawley rats, were purchased from Guangdong Medical Laboratory Animal Center. The animal experiments were carried out under the permission of the Shenzhen Advanced Animal Study Service Center (Approval No. AASC200615M). We complied with all relevant ethical regulations in the *in vivo* studies.

Synthesis of LOTUS

In a 1 mL 1-decanol solution, 1 g of LM (Pure Gallium) was introduced and subjected to sonication using a probe sonicator for 1 minute at 25% power (550 W) to transform the LM into LMPs. The resulting LMPs were isolated via centrifugation at 8000 rpm

for 5 minutes, and the supernatant was removed. The obtained LMPs were then transferred into the DMF containing TCPP (Tetrakis (4-carboxyphenyl) porphyrin) and subsequently subjected to sonication for 1 minute at 20% power (550 W) to initiate the preliminary modification of TCPP on the LMPs. The sonicated solution was further heated to 100 ^{o}C and stirred with a magnetic bead for 10 hours.

Purification of LOTUS

After the synthesis reaction, the raw LOTUS product was subjected to a rigorous purification process to remove the DMF solvent and other impurities. The product was collected by centrifugation and sequentially washed as follows:

DMF Washing: Washed twice with fresh DMF to remove unreacted TCPP and other soluble by-products.

Ethanol Washing: Washed three times with absolute ethanol to thoroughly replace and remove the residual DMF solvent.

Dialysis: The product was then redispersed in deionized water and transferred to a dialysis bag (MWCO: 14 kDa). It was dialyzed against deionized water for 7 days, with the water changed twice daily, to ensure complete removal of any residual organic solvents and obtain a biocompatible material for subsequent biological experiments.

The effectiveness of DMF removal was confirmed by Fourier-transform infrared (FTIR) spectroscopy (**Fig. S3**), which showed no characteristic peaks of DMF in the purified LOTUS sample.

***In vitro* antibacterial test**

For the *in vitro* antibacterial test, six bacterial strains were selected to evaluate the antibacterial activity of LOTUS, including laboratory-sensitive strains (ATCC) and clinically isolated MDR strains: *Pseudomonas aeruginosa*, *Staphylococcus aureus*, *Staphylococcus epidermidis*, *Escherichia coli*, *MRSA*, and *MRSE*. For the assay, bacterial suspensions were diluted to a final concentration of 10^5 CFU/mL and 50 μL of each suspension was transferred to a 96-well plate.

To comprehensively evaluate the contribution of each component, the following control groups were included alongside the LOTUS experimental group: (1) pure TCPP ligand, (2) pure LMPs, and (3) a physical mixture of TCPP and LMPs (TCPP+LMPs, 1:1 mass ratio). Stock solutions of TCPP, LMPs, and TCPP+LMPs were prepared in phosphate-buffered saline (PBS) at a concentration of 500 μg/mL. The stock concentration of the LOTUS composite was determined via inductively coupled plasma optical emission spectrometry. This measured stock was then appropriately diluted to obtain the working solution (concentration: 153.6 μg/mL). The MIC was determined using a standard two-fold serial dilution method in 96-well plates. Each bacterial strain and material combination was tested in triplicate.
To assess the impact of light exposure, the plates were subjected to visible light irradiation for 30 minutes before being incubated at 37 ^{o}C in a bacteriological incubator. After incubation, the plates were covered with aluminum foil to prevent any further light exposure. The MIC for each bacterial strain was determined following overnight incubation.

Observation of LOTUS acted bacterial morphologies

The morphologies of bacteria, both prior to and following treatment with LOTUS, were examined using scanning electron microscopy (SEM). Bacterial samples were fixed in a 2.5% glutaraldehyde solution and incubated at 4 ^{o}C for 24 hours. Subsequently, the samples underwent sequential dehydration with a gradient of ethanol concentrations (30%, 50%, 70%, 80%, 90%, and 100%), each step lasting 15 minutes. The dehydration process was conducted with meticulous care and at a gradual pace to preserve the native bacterial morphology, thereby minimizing the risk of inducing extraneous damage.

Detection of bacterial viability:

To assess bacterial viability, fluorescence images were acquired using a confocal microscope (Nikon A1R, Japan). Bacterial cells, either treated with LOTUS or left untreated as controls, were harvested via centrifugation and washed three times with

phosphate-buffered saline (PBS) to remove residual LB medium. The cells were then stained with a PBS solution containing propidium iodide (PI) and SYTO9 dyes for 30 minutes. Aliquots of the resulting suspensions were deposited onto microscope slides and allowed to settle prior to imaging.

Biocompatibility assessment of LOTUS

The biocompatibility of LOTUS with respect to erythrocytes was evaluated using fresh blood samples obtained from rats. The blood was centrifuged at 1,500 rpm for 15 minutes, and the resulting erythrocytes were washed three times with saline to remove plasma and other components. The erythrocytes were then resuspended to form a 4% (v/v) suspension. These suspensions were combined with LOTUS at varying concentrations and incubated at 37 °C for 3 hours. Deionized water was used as a positive control to induce hemolysis. Following incubation, the supernatants were collected by centrifugation at 12,000 rpm for 15 minutes, and their absorbance at 540 nm was measured using a UV–Vis spectrophotometer to confirm the absence of significant hemolysis.

Cell viability assays were performed to further assess the biocompatibility of LOTUS by culturing human umbilical vein endothelial cells (HUVECs; ATCC, USA) with LOTUS at concentrations ranging from 0 to 8 times the MIC. HUVECs were seeded at a density of 10^5 cells mL^{-1} in 6-well plates and maintained in Dulbecco's Modified Eagle Medium (DMEM) supplemented with 10% fetal bovine serum (FBS) under standard conditions (5% CO_2, 37 °C). For imaging, the cells were stained with calcein-AM (Macklin, China) and propidium iodide (PI; Macklin, China) for 20 minutes. Nuclear staining was conducted using a DAPI solution (Sigma, USA; 1:1000 dilution in PBS), followed by three washes with PBS. Fluorescent images were captured using a confocal laser scanning microscope (Nikon A1R, Japan) to verify cell viability and compatibility with LOTUS.

***In Vivo* assessment using rat wound models**

Wound models were established by creating cutaneous wounds on the dorsal surface of Sprague-Dawley (SD) rats (female, approximately 250 g), selected as the model animals to evaluate the *in vivo* antibacterial performance of LOTUS. The rats were randomly assigned to two groups and infected with either MRSA or S. aureus, respectively. Three wounds of uniform size (diameter ≈ 15 mm) were created on each rat. The wounds were inoculated with 200 μL of bacterial suspension (10^8 CFU mL^{-1}) of either MRSA or S. aureus and exposed for 30 minutes to ensure effective infection. Subsequently, 200 μL of LOTUS solution or phosphate-buffered saline (PBS) was applied to the wounds. No additional light irradiation was employed. Wound progression was monitored, and photographs were taken at intervals of 0, 3, 6, and 14 days post-treatment.

For microbiological analysis, wound surfaces were sampled using sterile swabs, which were then immersed in PBS. The resulting suspensions were subjected to bacterial enumeration via the spread plate method. On day 14, the rats were euthanized, and wound tissues were harvested and fixed in 4% paraformaldehyde. For histological evaluation, the fixed tissues were processed, embedded in paraffin, and sectioned. Hematoxylin and eosin (H&E) staining was performed by deparaffinizing the sections, staining with hematoxylin and eosin Y, and dehydrating with absolute ethanol. For Masson's trichrome staining, deparaffinized sections were sequentially treated with potassium dichromate, Weigert's iron hematoxylin, Biebrich scarlet-acid fuchsin, phosphomolybdic/phosphotungstic acid, and aniline blue solutions. The sections were then immersed in acetic acid for several minutes before being mounted. Images of stained sections were acquired using a bright-field microscope (Nikon Eclipse E100) for subsequent analysis. The mouse ear vascular network was imaged using a multi-scale photoacoustic/ultrasound imaging platform modified from a commercial multi-modality photoacoustic microscope (FPAM-50, PAOMTek Inc.).

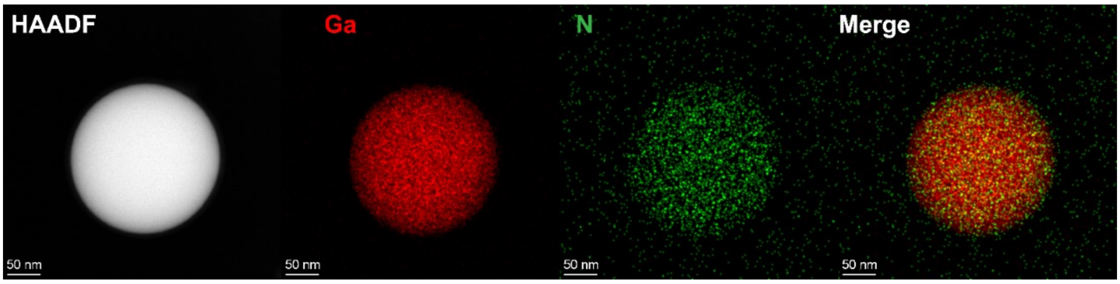

Fig. S1 TEM EDS images of LMPs sonicated with TCPP.

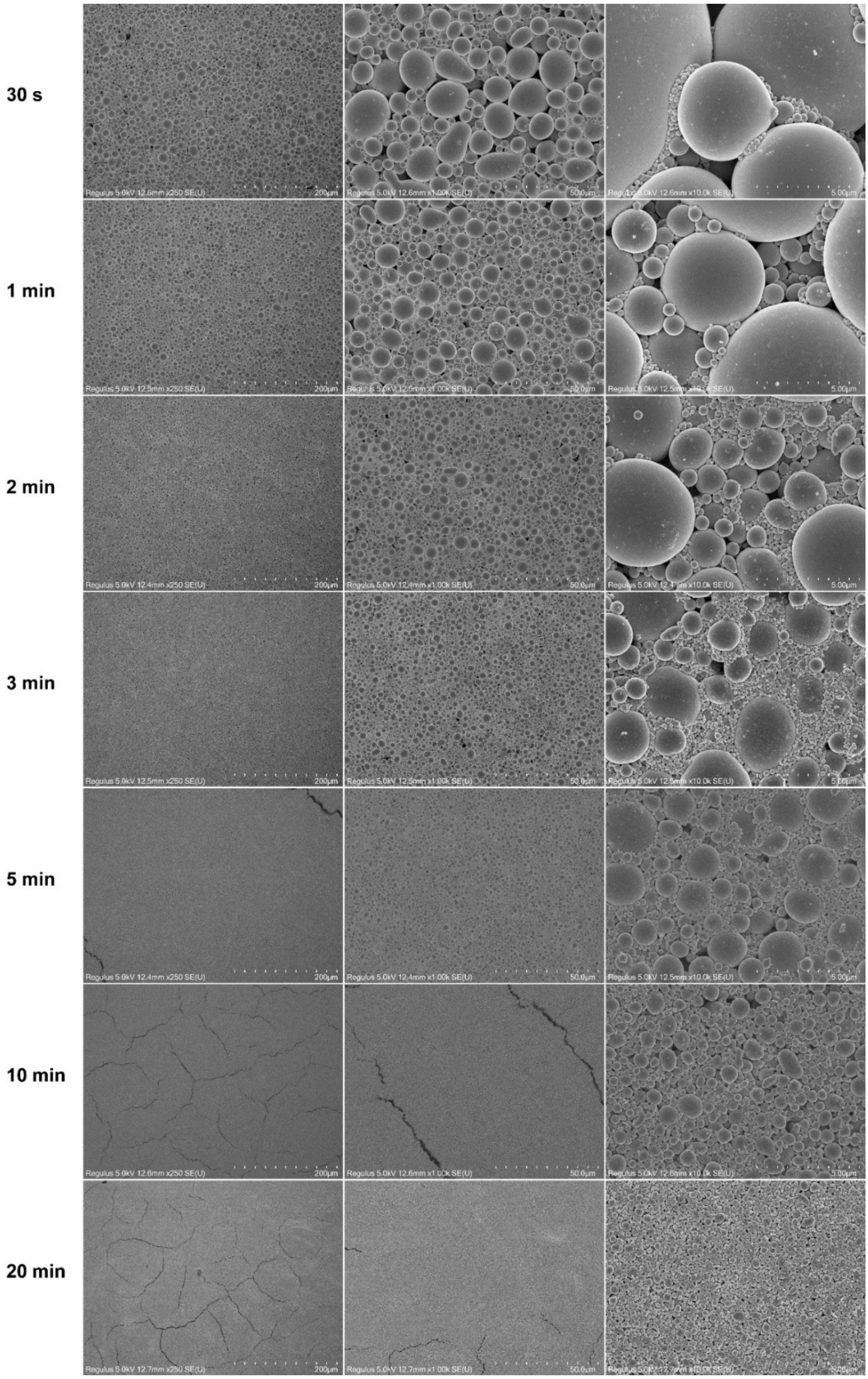

Fig. S2 SEM images of LMPs with different sonication time.

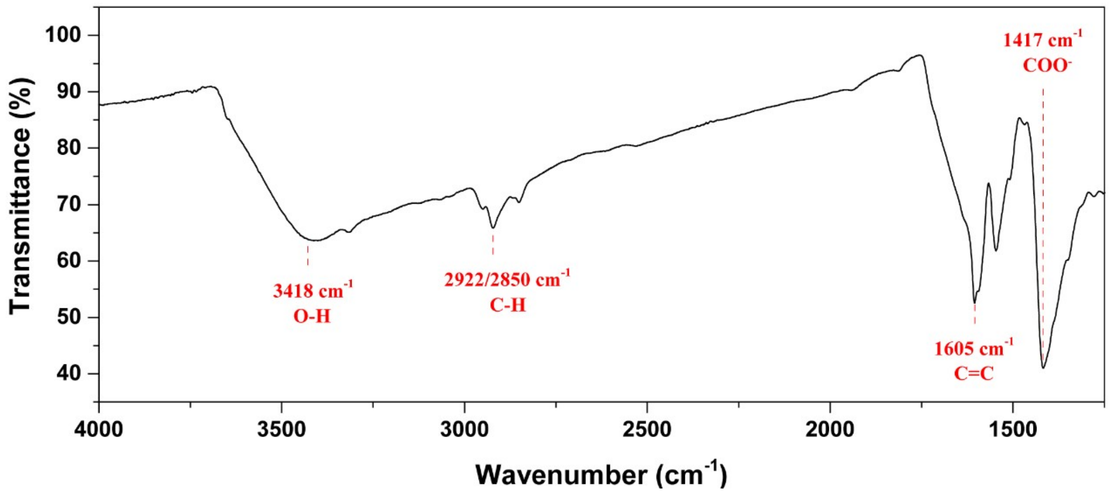

Fig. S3 Fourier Transform infrared spectroscopy (FTIR) of LOTUS.

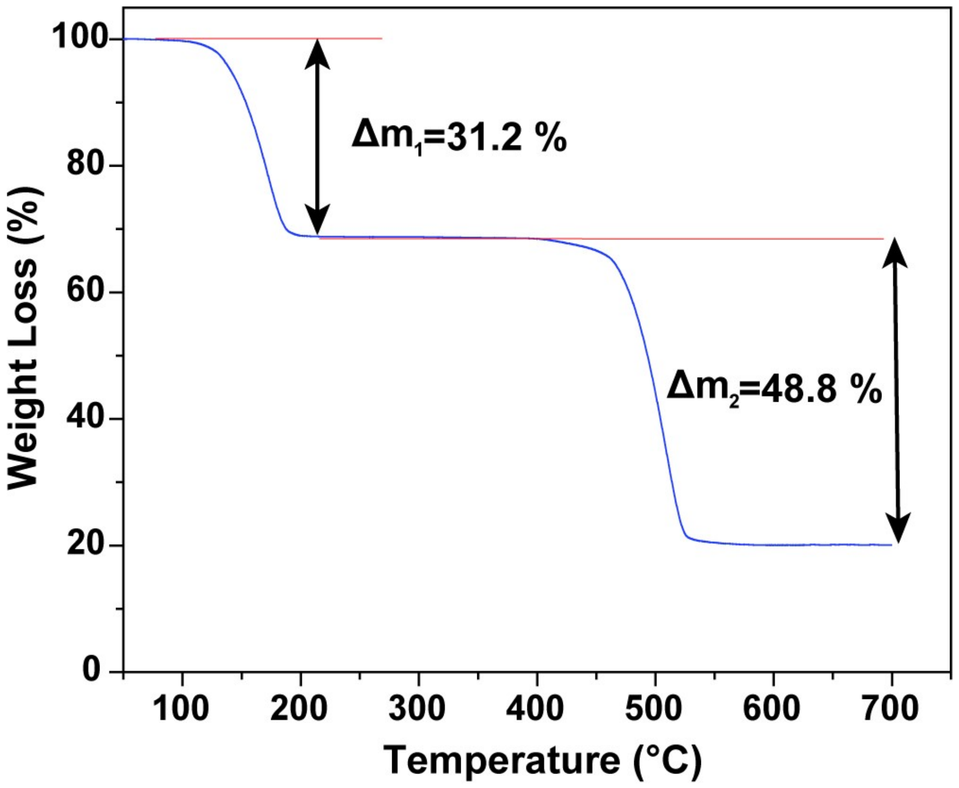

Fig. S4 Thermogravimetric Analysis (TGA) of LOTUS.

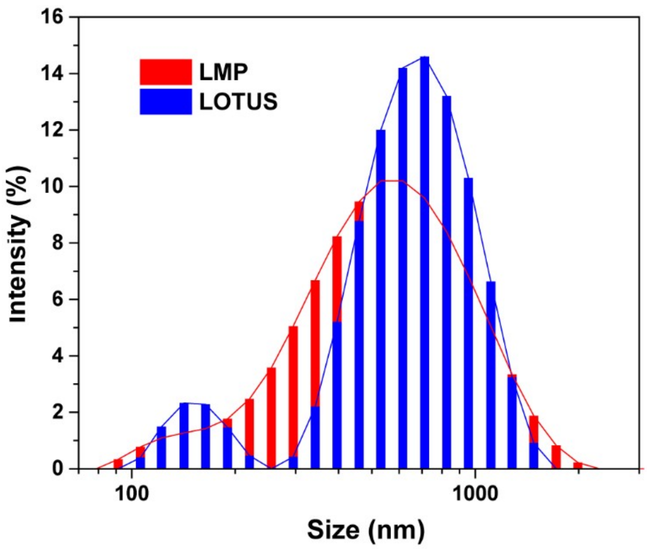

Fig. S5 Dynamic Light Scattering (DLS) of LOTUS.

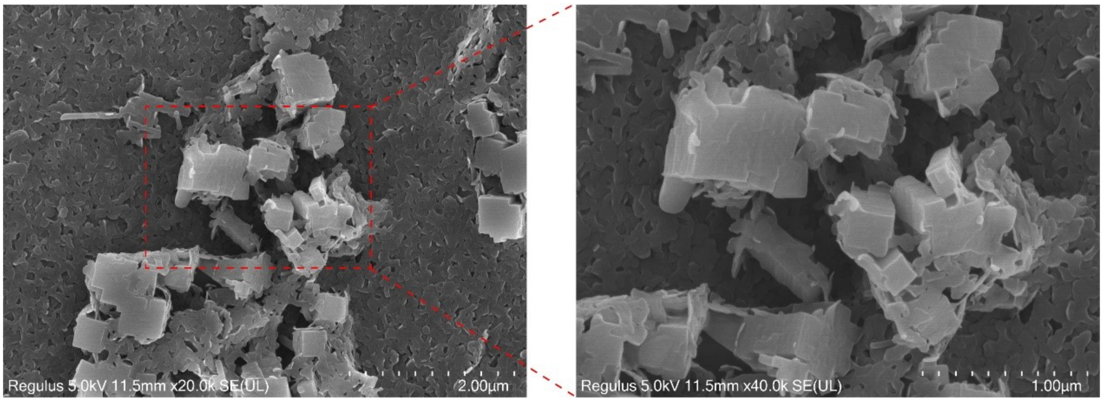

Fig. S6 Morphology of conventionally synthesized Ga-TCPP MOFs.

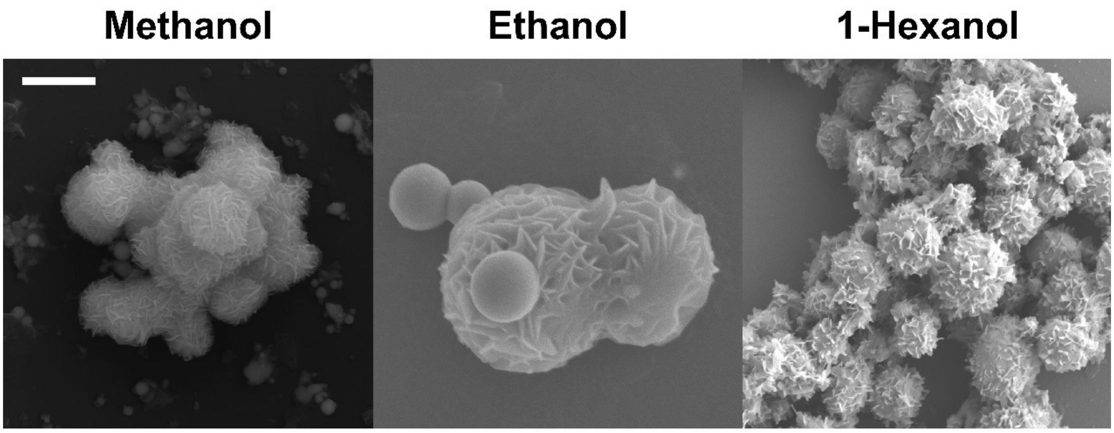

Fig. S7 Comparison of LOTUS synthesized using different alcohols in the pre-sonication step, the scale bar is 5 um.

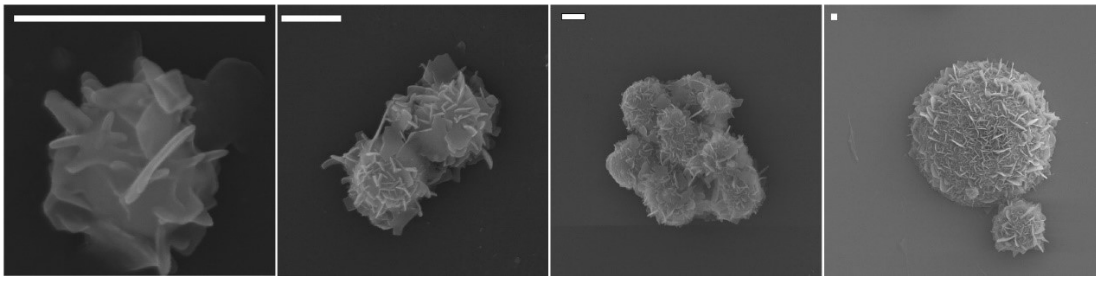

Fig. S8 Morphology of LOTUS synthesized on LMPs of different sizes, the scale bar is 500 nm.

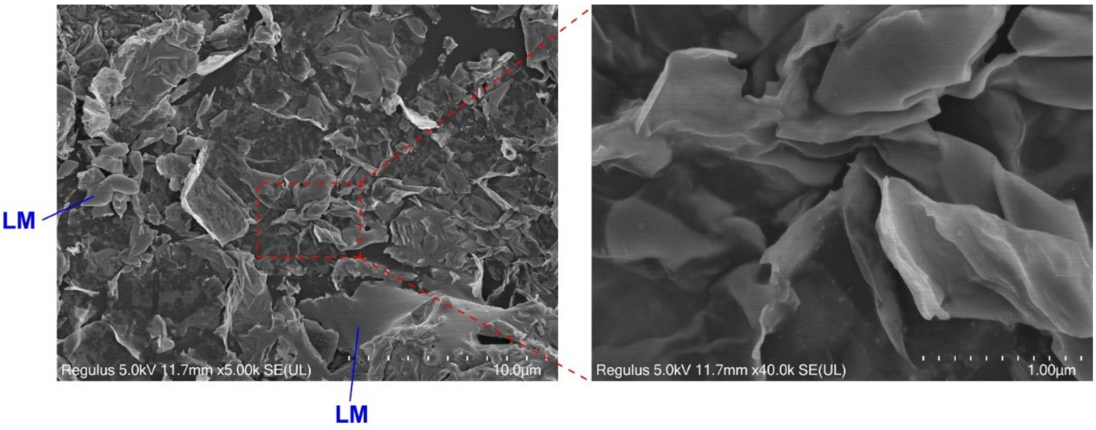

Fig. S9 Synthesis of Ga-TCPP on a flattened liquid metal film.

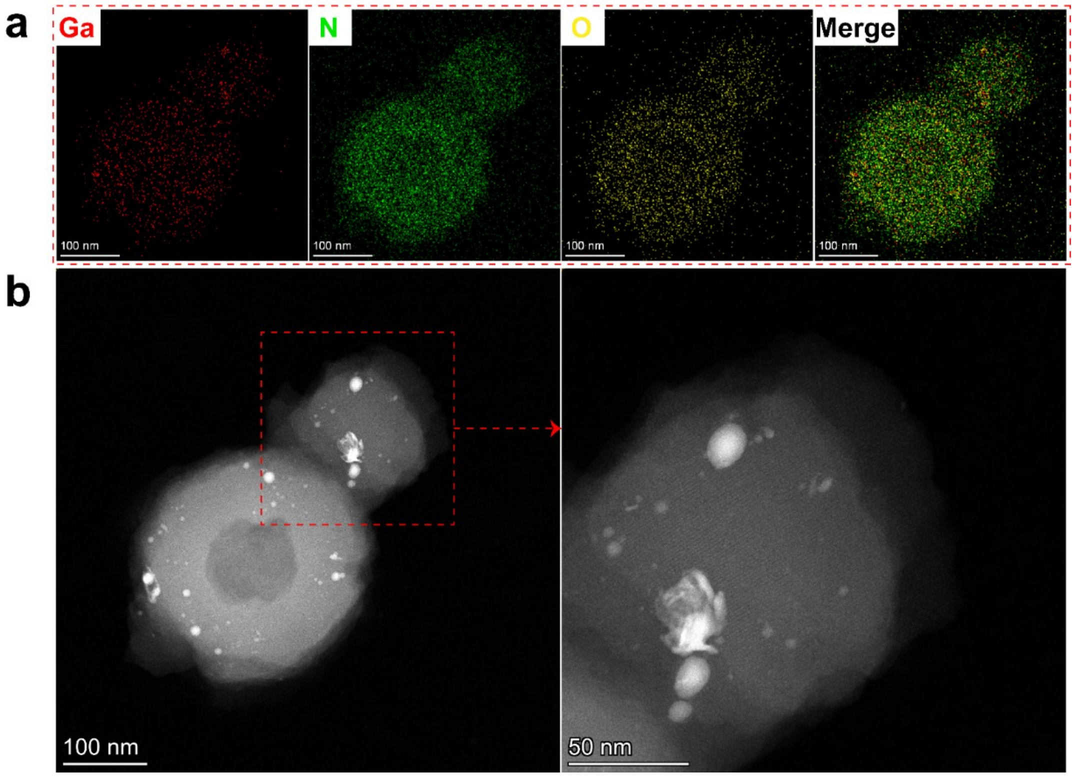

Fig. S10 TEM images of LOTUS treated with LB medium overnight. a.

TEM EDS of the LOTUS; b. TEM image of LOTUS and the enlarged image of the area in dashed box.

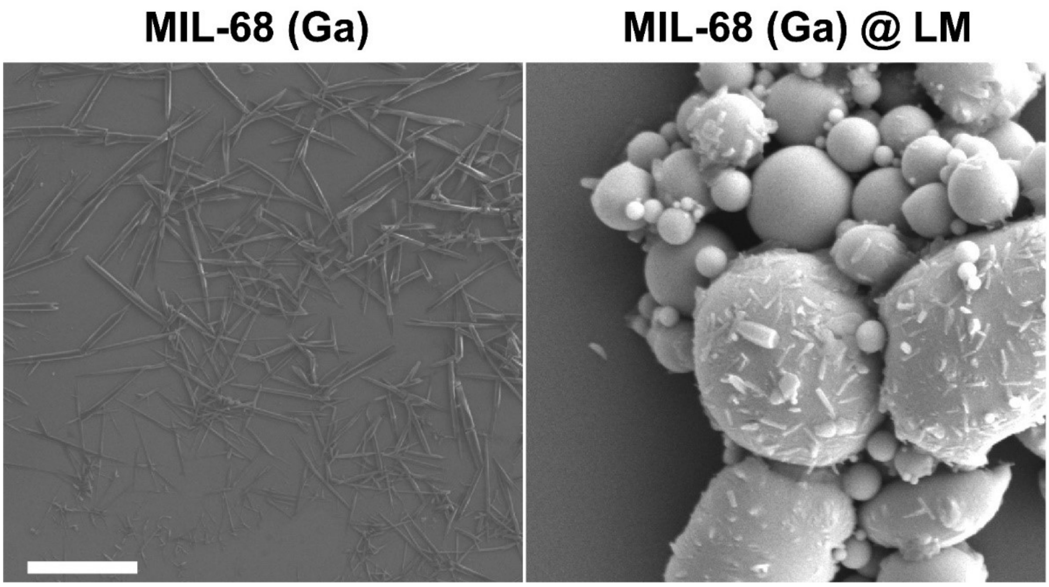

Fig. S11 Morphological comparison of a 3D Ga-MOF synthesized under conventional.

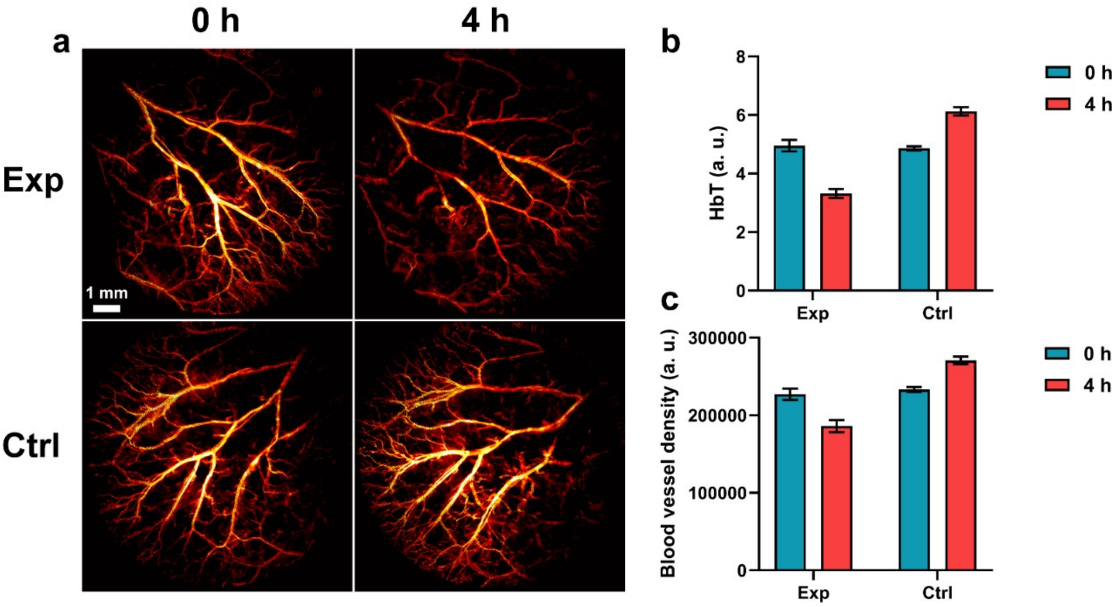

Fig. S12 In vivo evaluation of LOTUS's antibacterial efficacy and injection safety using photoacoustic imaging in a mouse ear infection model. (a) Photoacoustic imaging of ear for the experiment (injection of

10 μL S. aureus [10^5 per CFU] and 10 μL LOTUS [1MIC]) and control (injection of 10 μL S. aureus [10^5 per CFU] and 10 μL PBS) group before and after the injection. (b) Hemoglobin concentration in the ears of the control and experiment group. (c) Blood vessel density in the ears of the control and experiment group. Data presented as mean ± SD (n=5).
